# Determinants of malnutrition in older hospitalized patients: a prospective multicenter study with the DoMAP model

**DOI:** 10.1186/s12877-026-07612-6

**Published:** 2026-05-07

**Authors:** Maryam Pourhassan, Stefan Pfannkuch, Kiril Stoev, Maria Schnepper, Isabel Levermann, Baigang Wang, Chantal Giehl, Ulrike Trampisch, Lukas Funk, Ilse Gehrke, Wolfried Schröer, Dorothee Volkert, Rainer Wirth

**Affiliations:** 1 Department of Geriatric Medicine, Marien Hospital Herne, University-Hospital of Ruhr-University Bochum, Hölkeskampring 40, 44625 Herne, Germany; 2 Department of Geriatric Medicine IV, Klinikverbund Südwest, Böblingen, Germany; 3 Department of Geriatric Medicine, Sana Hospital, Duisburg, Germany; 4 Institute for Biomedicine of Aging, Friedrich-Alexander-Universität Erlangen-Nürnberg, Nuremberg, Germany

**Keywords:** Malnutrition, DoMAP model, GLIM criteria, Older adults, Diseases, Symptoms

## Abstract

**Background:**

Few studies have assessed the full spectrum of potential causes of malnutrition in older hospitalized patients using a standardized framework. Applying the “Determinants of Malnutrition in Aged Persons” (DoMAP) model, this study aimed to quantify the prevalence of specified determinants and compare their distribution between malnourished and non-malnourished patients, thereby identifying the most important determinants.

**Methods:**

This multicenter, cross-sectional, prospective, observational study was conducted in three geriatric acute care hospital units. Malnutrition was diagnosed using the Global Leadership Initiative on Malnutrition (GLIM) criteria. Potential causes of malnutrition were assessed using the DoMAP model. This study was registered in the German Clinical Trials Register with the DRKS-ID: DRKS00030850 on December 14, 2022.

**Results:**

A total of 556 patients (mean age 82.5 ± 6.6 years; 67% women) were included. Malnourished patients exhibited a significantly higher prevalence of most determinants compared to non-malnourished patients, particularly low intake (89 vs. 49%), poor appetite (68 vs. 25%), inflammation (31 vs. 19%), gastrointestinal disease (29 vs. 11%), inflammatory disease (32 vs. 19%), and hospitalization (62 vs. 47%). The mean total determinants count was significantly higher in malnourished participants (12.5 ± 5.3) than in non-malnourished ones (10.1 ± 4.0; *p* < 0.001). Regression analysis revealed low intake as the strongest determinant at Level1; poor appetite, at Level2; gastrointestinal disease and oral pain at Level3, and anorexia of aging and hospitalization at Level4.

**Conclusion:**

The DoMAP model provides a structured framework for capturing the diverse etiologies of malnutrition in older patients. This study emphasizes the multifactorial nature of malnutrition in hospitalized patients, with low intake and poor appetite emerging as predominant drivers.

**Supplementary Information:**

The online version contains supplementary material available at 10.1186/s12877-026-07612-6.

## Introduction

Malnutrition is a common and serious condition among older hospitalized adults, associated with adverse clinical outcomes including prolonged hospital stay, increased readmission rate, functional decline, and higher mortality risk [1, 2]. Older inpatients often present with multimorbidity, polypharmacy and geriatric syndromes, such as cognitive impairment, and functional limitations, all of which increase the risk for nutritional deterioration [3]. Insufficient dietary intake appears to be the key contributor to this decline, often driven by age-associated physiological changes, underlying illnesses, and environmental limitations. Additionally, elevated metabolic demands due to acute and chronic diseases, psychological distress, and limited socioeconomic resources can impair nutritional balance and accelerate the development of malnutrition in this population [2, 4].

While malnutrition in hospitalized settings is frequently underdiagnosed and undertreated, studies have shown that 30–50% of older inpatients may be malnourished or at risk, depending on the population and criteria used [5–7]. Multiple interrelated factors may contribute to the development of malnutrition in older hospitalized patients [8, 9], with varying effects across individuals. Given the complex and multifactorial nature of malnutrition in this population, a comprehensive and structured approach to identify its underlying causes is crucial. Accurate diagnosis enables the timely initiation of personalized nutritional interventions, which are essential to prevent further deterioration and improve clinical outcomes. Furthermore, systematic assessment of relevant risk factors is critical for guiding healthcare teams in the delivery of targeted, effective care. Even though not all individual causes of malnutrition may be modifiable, their improvement and compensation should be part of an individualized treatment concept.

However, despite its importance, few studies have assessed the full spectrum of potential causes and malnutrition-related determinants using a standardized framework. The “Determinants of Malnutrition in Aged Persons” (DoMAP) model — developed through a multidisciplinary European expert consensus — offers such a framework by organizing risk factors into four interrelated levels, ranging from core pathophysiological mechanisms (e.g., low intake) to broader contextual contributors (e.g., frailty, polypharmacy, and hospitalization) [9].

Using the DoMAP framework, this study aimed to quantify the prevalence of its specified malnutrition causes and to compare their distribution between malnourished and non‐malnourished older hospitalized patients, thereby identifying the most important determinants assessed at or shortly after hospital admission.

## Subjects and methods

This multicenter, cross-sectional, prospective, observational study was conducted between December 2022 and March 2024 in three geriatric acute care hospital units. A total of 556 patients were consecutively enrolled during their hospital stay. Eligible participants were aged 75 years or older and had available and reliable body weight data. Exclusion criteria comprised terminal illness, clinically relevant fluid imbalance (such as decompensated heart failure or symptomatic dehydration), hemodialysis, enteral or parenteral nutrition for more than two weeks, and limb amputation(s).

All participants meeting the inclusion criteria were approached by a trained study physician, who provided both verbal and written information regarding the study objectives, procedures, and data handling. Informed consent was obtained in accordance with ethical standards.

### Assessment and definition of malnutrition

Malnutrition was diagnosed based on the Global Leadership Initiative on Malnutrition (GLIM) criteria [10, 11]. All patients initially underwent screening using the Mini Nutritional Assessment Short Form (MNA-SF) [12] with a total possible score of 14 points. Scores of 8–11 indicate a risk of malnutrition, while scores below 8 classify individuals as malnourished. However, the GLIM criteria were applied independently of the screening results to ensure an objective assessment of nutritional status according to GLIM and for being able to compare both tools in a later analysis. GLIM defines malnutrition using a combination of phenotypic and etiologic criteria. The phenotypic components include (1) non-voluntary weight loss of more than 5% within the past six months or > 10% beyond six months, (2) a body mass index (BMI) below 22 kg/m^2^ for persons ≥ 70 years, and (3) reduced muscle mass. In this study, only weight loss and BMI were used for phenotypic assessment. Etiologic criteria comprised (1) reduced food intake defined as intake below 50% of energy requirements during the previous week, or any sustained reduction for more than two weeks, (2) the presence of gastrointestinal conditions that impair nutrient assimilation or absorption, and (3) evidence of inflammation. Based on the guidance for assessment of the inflammation etiologic criterion for the GLIM diagnosis of malnutrition [13], inflammation was assessed based on clinical judgement, integrating diagnoses and clinical presentation of acute or chronic inflammatory conditions. Laboratory parameters, including C-reactive protein (CRP), were used as supportive evidence, particularly in cases of uncertainty. No fixed CRP cut-off was applied.

In addition, patients were asked to quantify the extent and timing of weight loss, categorized into intervals of three months, six months, and beyond six months.

### Assessment of malnutrition determinants

**Fig. 1:**
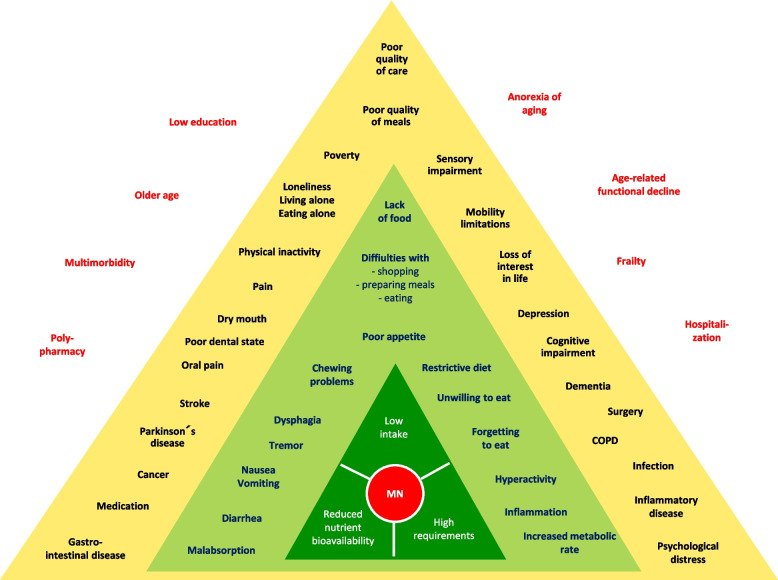
Determinants of Malnutrition in Aged Persons (DoMAP) model

Malnutrition determinants were assessed using the Determinants of Malnutrition in Aged Persons (DoMAP) model—a structured conceptual framework to harmonize the identification and understanding of malnutrition drivers in older populations [9]. The model is organized into three interrelated, concentric triangle-shaped levels, surrounded by some general factors and with malnutrition positioned centrally (Fig. 1).

Level 1 represents the core mechanisms of malnutrition, comprising low nutritional intake, increased nutritional requirements, and reduced nutrient bioavailability. Level 2 encompasses 16 direct contributing factors, such as poor appetite and dysphagia (impacting intake), diarrhea (reducing bioavailability), and elevated metabolic demand (increasing requirements). Level 3 includes 25 indirect contributing factors that influence nutritional status through one or more Level 2 mechanisms—for example, stroke, which may cause dysphagia and impair intake, or medications that may induce nausea or vomiting, thereby impairing intake and absorption. Level 4 (surrounding/contextual factors) captures broader age-related and socio-medical risks with a long-term, indirect effect on nutritional status. These include polypharmacy, multimorbidity, frailty, low educational level, anorexia of aging, and recent hospitalization.

To ensure consistency in data collection, standard definitions were provided for items not self-explanatory. Multimorbidity was defined as the presence of three or more chronic diseases; polypharmacy on admission as regular intake of five or more medications (excluding vitamins and minerals). Anorexia of aging was defined as appetite reduction without identifiable pathology. Age-related functional decline referred to functional loss not attributable to a single disease. Hospitalization was defined as any hospital admission within the past 12 months except the present hospitalization. Stroke included active or residual stroke conditions. Cancer was defined as an active malignant disease. Surgery referred to any operative procedure performed within the preceding three months. Medication-related side effects were recorded if adverse effects were suspected to impact appetite, digestion, or nutrient absorption. Restrictive diet was defined as adherence to a prescribed or self-imposed diet that significantly limits energy, carbohydrate, protein or fat intake. Sensory impairment was defined as gustatory, olfactory, visual or auditory impairment with impact on everyday life. In addition, increased nutritional requirements were defined as a clinically judged increase in nutritional needs beyond normal requirements due to acute or chronic disease-related catabolic or stress-related conditions, such as infection, inflammation, fever, or wound healing. Low nutritional intake was defined as any clinically relevant reduction in food intake, without applying a fixed quantitative cut-off.

The assessment of DoMAP determinants was performed by the attending physician. The DoMAP framework was used as a structured checklist during the hospital stay. After several days of clinical observation and review of the patient’s medical history, nursing documentation, laboratory parameters, and, where applicable, information provided by patients or relatives, the physician documented which determinants were present. The final classification of determinants was therefore based on clinical judgement integrating all available sources of information.

### Geriatric assessment

At hospital admission, all patients underwent a standardized comprehensive geriatric assessment (CGA) including frailty, functional ability, cognitive status, mood, and nutritional status. Frailty was assessed using the Clinical Frailty Scale (CFS), which classifies individuals on a scale from 1 (very fit) to 9 (terminally ill); a score of ≥ 6 was used to define frailty [14]. Nutritional status was assessed using the MNA-SF [12]; this instrument was also used as the initial screening step within the GLIM diagnostic process (as described above). Functional dependence was assessed via the Barthel Index, with a point range from 0 to 100 points, with scores of 40–55 indicating moderate dependence, 20–35 indicating severe dependence, and scores below 20 pointing to very severe dependence [15]. Cognitive function was evaluated using either the Montreal Cognitive Assessment (MoCA) [16] or other local standards. A MoCA score below 26 were considered indicative of cognitive impairment. Depressive symptoms were assessed utilizing the Depression in Old Age Scale (DiA-S) [17] or the Geriatric Depression Scale (GDS) [18]. DiA-S categorizes depression as no (0—2 points), suspected (3 points), or probable (4—10 points) and GDS-15 allows for classification into three categories: normal (0—5 points), mild to moderate (6—10 points), and severe depressive symptoms (11—15 points).

### Statistical analysis

Statistical analyses were performed using SPSS Statistics for Windows (Version 29.0, IBM Corp, Armonk, NY, USA).

Due to limited preliminary data to inform a definitive sample size calculation, assumptions were made based on low-prevalence determinants. For instance, estimating the prevalence of Parkinson’s disease as 2% in non-malnourished patients and 6% in malnourished patients, with a two-sided Fisher’s exact test and α = 0.05, yielded a required sample size of *n* = 425. To allow for adequate subgroup differentiation and account for potential dropouts, a minimum target of 500 patients was set for enrolment.

Continuous variables are expressed as means and standard deviations (SD) for normally distributed data, or medians and interquartile ranges (IQR) for non-normally distributed data. Categorical variables are reported as absolute frequencies and percentages. Group comparisons between malnourished and non-malnourished patients were performed using the Pearson Chi-square test for categorical variables.

To assess the cumulative burden of risk factors, the number of each patient’s determinants (based on the DoMAP model) were summed to calculate a total determinant count, along with level-specific counts. These were compared between malnourished and non-malnourished patients using independent samples t-tests.

To explore possible collinearity and interrelationships across the DoMAP levels, partial correlation analyses were conducted to determine the degree of association between determinants within and across levels. Finally, binary logistic regression analyses were carried out separately for each DoMAP level, with malnutrition status (yes/no) as the dependent variable. Independent variables were the relevant determinants within each level. Regression outcomes are reported as regression coefficients (B), standard errors (SE), odds ratios (Exp(B)) with 95% confidence intervals (CI), and *p*-values. A two-sided *p*-value < 0.05 was considered statistically significant.

## Results

Baseline characteristics of the study population, stratified by malnutrition status according to the GLIM criteria, are summarized in Table 1.

**Table 1:** Characteristic of study population on admission

	All (*n* = 556)	*Non-malnourished (*n* = 319, 57%)	Malnourished (*n* = 237, 43%)	**P value
Gender				
Female, n (%)	370 (67)	211 (66)	159 (67)	0.856
Male, n (%)	186 (33)	108 (34)	78 (33)	
Age (y), mean ± SD	82.5 ± 6.6	83.1 ± 6.3	81.7 ± 6.9	0.017
Height (m), mean ± SD	1.65 ± 0.09	1.65 ± 0.09	1.65 ± 0.09	0.776
Body weight (kg), mean ± SD	72.2 ± 17.5	78.6 ± 17.0	63.7 ± 14.2	< 0.001
BMI (kg/m^2^), mean ± SD	26.2 ± 5.9	28.5 ± 5.7	23.1 ± 4.6	< 0.001
Previous weight loss				
No, n (%)	289 (52)	252 (79)	37 (16)	< 0.001
Yes, n (%)	267 (48)	67 (21)	200 (84)	
Previous weight loss (kg), mean ± SD	3.7 ± 6.1	0.7 ± 2.1	7.8 ± 7.5	< 0.001
Duration of previous weight loss (m)				
< 3 months, n (%)	154 (28)	48 (15)	106 (45)	< 0.001
< 6 months, n (%)	41 (7)	7 (2)	34 (14)	
> 6 months, n (%)	72 (13)	12 (6)	60 (25)	
Total of total determinants, mean ± SD	11.1 ± 4.8	10.1 ± 4.0	12.5 ± 5.3	< 0.001
At level 1 of DoMAP model	0.8 ± 0.7	0.6 ± 0.6	1.2 ± 0.7	< 0.001
At level 2 of DoMAP model	1.8 ± 1.7	1.3 ± 1.3	2.5 ± 2.0	< 0.001
At level 3 of DoMAP model	5.5 ± 2.7	5.4 ± 2.5	5.7 ± 2.8	0.184
At level 4 of DoMAP model	2.9 ± 1.2	2.7 ± 1.2	3.1 ± 1.3	< 0.001
Geriatric assessment				
MNA-SF, median (IQR)	8 (6—10)	9 (8—10)	6 (5—8)	< 0.001
Normal nutritional status, n (%)	34 (6)	33 (10)	1 (0)	< 0.001
Risk of malnutrition, n (%)	284 (51)	221 (69)	63 (27)	
Malnourished, n (%)	238 (43)	65 (21)	173 (73)	
Barthel-Index, median (IQR)	50 (40—65)	50 (45—65)	50 (40—60)	< 0.001
Cognitive Testing				
MoCA, Median (IQR)	18 (15—21)	18 (15—21)	17 (14—20)	0.070
Depression Testing				
DiA, n (%)	499 (91)	280 (88)	219 (94)	
Median (IQR)	3 (1—5)	3 (1—5)	3 (1—5)	0.404
GDS, n (%)	52 (9)	37 (12)	15 (6)	
Median (IQR)	3 (2—6)	3 (2—6)	2 (2—5)	0.470
Clinical Frailty Scale, median (IQR)	5 (5—6)	5 (5—6)	6 (5—6)	
Frailty, n (%)	277 (50)	170 (53)	109 (46)	0.103
No Frailty, n (%)	279 (50)	149 (47)	128 (54)	

*MNA-SF* Mini Nutritional Assessment Short Form

^*^Malnutrition was diagnosed based on the Global Leadership Initiative on Malnutrition (GLIM) criteria

**Difference between malnourished and non-malnourished participants

A total of 556 patients were included in the analysis. Major reasons for hospital admission included falls, fractures, infections, heart failure and neurological diseases such as stroke and dementia. Based on the GLIM criteria, 43% of patients were classified as malnourished.

The mean age was 82.5 ± 6.6 years, and 67% were women. Nearly half of the patients (48%) reported unintentional weight loss prior to admission, with a mean weight loss of 3.7 ± 6.1 kg in the entire population and 7.8 ± 7.5 kg in those with weight loss. Among them, 28% had lost weight within the last 3 months, 7% within 6 months, and 13% for more than 6 months.

The mean total number of malnutrition determinants was 11.1 ± 4.8 in the overall study population, and 50% of the patients were identified as frail. The median MNA-SF score was 8; 6% were classified as having normal nutritional status, 51% at risk of malnutrition, and 43% as malnourished. The median Barthel Index was 50, reflecting considerable functional dependence. Cognitive testing was available for 427 patients (243 non-malnourished and 184 malnourished). The median MoCA score in total population was 18, with 94.1% (*n* = 402) scoring < 26 points, indicating cognitive impairment.

Psychological assessment using the DiA-S (*n* = 499) showed a median score of 3, with 49% (*n* = 169) classified as having probable depression and 15% (*n* = 73) as having suspected depression. Among those assessed with the GDS-15 (*n* = 52), 22% (*n* = 11) and 4% (*n* = 2) exhibited mild-to-moderate and severe depressive symptoms, respectively.

Significant differences between malnourished and non-malnourished patients were observed for all baseline variables except gender, height, cognitive function, depressive symptoms, and frailty.

**Fig. 2:**
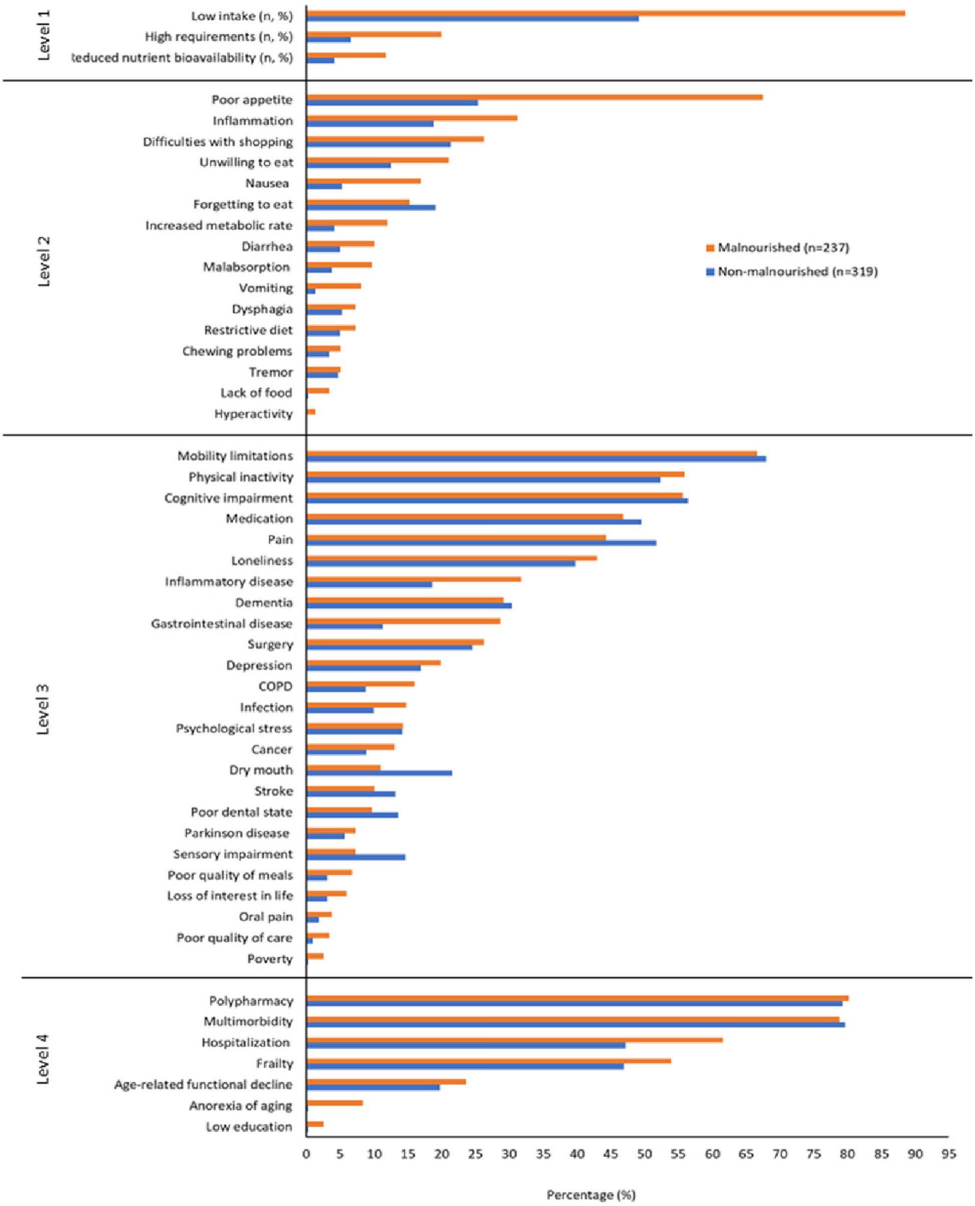
Prevalence and comparison of malnutrition determinants based on the DoMAP model between malnourished (*n* = 237) and non-malnourished (*n* = 319) older hospitalized patients. Bars show the percentage of participants affected by each determinant, grouped according to the four DoMAP levels (Level 1: core mechanisms; Level 2: direct determinants; Level 3: indirect determinants; Level 4: contextual factors)

Figure 2 provides a visual summary of the prevalence of malnutrition determinants across all four levels of the DoMAP model, comparing malnourished and non-malnourished patients. In line with these visual findings, supplementary Tables 1–4 present detailed frequencies of individual determinants within the total cohort and stratified by malnutrition status based on the GLIM criteria. Supplementary Table 1 focuses on level 1 determinants of malnutrition within the DoMAP. Among malnourished patients, low nutritional intake was the most prevalent determinant, observed in 89% of cases, significantly higher than in non-malnourished patients (49%; *P* < 0.001). As expected, all three core mechanisms—low intake, high requirements, and reduced bioavailability—were significantly more frequent in malnourished individuals compared to their non-malnourished counterparts.

According to Fig. 2, among malnourished patients, poor appetite was the most prevalent Level 2 determinant, reported in 68% of cases, compared to 25% in non-malnourished individuals (*P* < 0.001). In addition, malabsorption, diarrhea, nausea, vomiting, lack of food, unwillingness to eat, inflammation, and increased metabolic rate were significantly more frequent in malnourished patients compared to their non-malnourished counterparts (all *P* < 0.05; Supplementary Table 2).

As illustrated in Fig. 2, mobility limitation was the most prevalent Level 3 determinant among malnourished patients (67%), yet its frequency did not differ significantly from that in non-malnourished individuals. In contrast, inflammatory disease emerged as the most frequent Level 3 factor showing significant differences between groups, alongside other conditions such as gastrointestinal disease, poverty and COPD. In contrast, dry mouth and sensory impairment were more prevalent among non-malnourished individuals (Supplementary Table 3).

Level 4 determinants in Fig. 2 show that among malnourished individuals, polypharmacy was the most prevalent condition (80%), although its prevalence did not differ significantly from that in non-malnourished patients. In contrast, prior hospitalization was the most prominent Level 4 factor showing significant group differences, alongside other less frequent but relevant factors such as low education and anorexia of aging (Supplementary Table 4).

The total number of malnutrition determinants was significantly higher among malnourished patients compared to their non-malnourished counterparts (Table 1). This difference remained significant across Levels 1, 2, and 4 of the DoMAP model (all *P* < 0.001), whereas no statistically significant difference was observed at Level 3 (*P* = 0.184).

Correlation analyses revealed significant interrelationships between determinants across different levels of the DoMAP model (for clarity, only correlations with coefficients > r = 0.3 are reported; all *P* < 0.001). For example, reduced nutrient bioavailability (Level 1) was strongly correlated with malabsorption (r = 0.659), diarrhea (r = 0.787), vomiting (r = 0.466), and nausea (r = 0.481). Low intake was significantly associated with poor appetite (r = 0.829), mobility limitations (r = 0.315), frailty (r = 0.317), and hospitalization (r = 0.310). Additionally, malabsorption and diarrhea correlated with gastrointestinal disease (r = 0.713 and r = 0.613, respectively). Poor appetite showed correlations with physical inactivity (r = 0.356), anorexia of aging (r = 0.331), and hospitalization (r = 0.314). Inflammation was associated with multimorbidity (r = 0.376) and polypharmacy (r = 0.330).

Binary logistic regression analysis (Table 2) identified multiple independent predictors of malnutrition across all four levels of the model. At Level 1, low intake emerged as the strongest predictor, followed by increased nutritional requirements. At Level 2, only poor appetite was significantly associated with malnutrition. At Level 3, significant predictors included gastrointestinal disease, oral pain, and inflammatory disease. In contrast, dry mouth, poor dental state, and sensory impairment were inversely associated with malnutrition. At Level 4, anorexia of aging and hospitalization within the past year showed significant positive associations with malnutrition.

**Table 2:** Binary logistic regression analysis of risk factors associated with malnutrition based on the DoMAP model

Levels of DoMAP model	*Malnutrition (yes/no)					
				95% CI for Exp(B)		
	B	Std. Error	Exp(B)	Lower	Upper	*P* value
Level 1						
Low intake	1.987	0.236	7.292	4.594	11.573	< 0.001
High requirements	0.915	0.303	2.497	1.378	4.524	0.003
Reduced nutrient bioavailability	0.727	0.382	2.069	0.978	4.376	0.057
Level 2						
Lack of food	1.898	1.215	6.672	0.617	72.174	0.118
Poor appetite	1.789	0.205	5.986	4.009	8.938	< 0.001
Hyperactivity	1.319	1.478	3.738	0.206	67.706	0.372
Vomiting	1.203	0.674	3.331	0.889	12.473	0.074
Malabsorption	0.869	0.456	2.384	0.976	5.822	0.057
Nausea	0.725	0.378	2.064	0.984	4.331	0.055
Increased metabolic rate	0.386	0.464	1.470	0.593	3.648	0.406
Difficulties with shopping	0.301	0.242	1.351	0.840	2.173	0.215
Inflammation	0.285	0.249	1.330	0.816	2.168	0.252
Chewing problems	0.211	0.539	1.235	0.429	3.554	0.696
Unwilling to eat	0.179	0.286	1.197	0.683	2.097	0.531
Forgetting to eat	0.122	0.278	1.130	0.655	1.950	0.661
Dysphagia	0.031	0.428	1.031	0.445	2.387	0.943
Tremor	−0.135	0.478	0.874	0.342	2.229	0.777
Restrictive diet	−0.163	0.470	0.849	0.338	2.133	0.728
Diarrhea	−0.223	0.453	0.800	0.329	1.945	0.622
Level 3						
Oral pain	1.293	0.624	3.645	1.074	12.373	0.038
Poverty	1.227	1.165	3.412	0.348	33.462	0.292
Gastrointestinal disease	1.201	0.239	3.325	2.081	5.312	< 0.001
Poor quality of care	1.108	0.796	3.028	0.637	14.400	0.164
Loss of interest in life	0.806	0.496	2.238	0.847	5.914	0.104
Inflammatory disease	0.625	0.224	1.868	1.204	2.898	0.005
COPD	0.448	0.286	1.565	0.894	2.740	0.117
Depression	0.371	0.256	1.448	0.877	2.393	0.148
Parkinson disease	0.228	0.360	1.256	0.620	2.541	0.527
Surgery	0.213	0.219	1.238	0.806	1.900	0.329
Cancer	0.186	0.294	1.204	0.676	2.145	0.528
Infection	0.180	0.283	1.198	0.688	2.084	0.524
Loneliness	0.142	0.198	1.152	0.782	1.699	0.473
Physical inactivity	0.107	0.199	1.113	0.754	1.642	0.590
Poor quality of meal	0.104	0.475	1.110	0.438	2.813	0.827
Dementia	0.073	0.250	1.076	0.659	1.757	0.770
Cognitive impairment	0.039	0.226	1.040	0.668	1.619	0.863
Psychological stress	0.025	0.273	1.025	0.600	1.751	0.928
Medication	−0.133	0.186	0.876	0.608	1.261	0.476
Mobility limitations	−0.150	0.208	0.861	0.572	1.295	0.472
Pain	−0.240	0.193	0.786	0.539	1.148	0.213
Stroke	−0.409	0.284	0.664	0.381	1.159	0.150
Poor dental state	−0.757	0.335	0.469	0.243	0.904	0.024
Dry mouth	−1.017	0.286	0.362	0.207	0.633	< 0.001
Sensory impairment	−1.064	0.338	0.345	0.178	0.669	0.002
Level 4						
Anorexia of aging	3.149	1.035	23.310	3.064	177.333	0.002
Low education	1.343	1.169	3.832	0.388	37.896	0.250
Hospitalization	0.564	0.185	1.757	1.222	2.526	0.002
Frailty	0.249	0.180	1.282	0.901	1.824	0.167
Age-related functional decline	0.038	0.226	1.038	0.667	1.617	0.868
Polypharmacy	−0.044	0.248	0.957	0.589	1.556	0.861
Multimorbidity	−0.212	0.248	0.809	0.498	1.316	0.393

^*^Malnutrition was diagnosed based on the Global Leadership Initiative on Malnutrition (GLIM) criteria

## Discussion

This multicenter study systematically applied the DoMAP framework to identify key determinants of malnutrition in older hospitalized adults. Our findings confirm the multifactorial nature of malnutrition and highlight several direct and indirect determinants across all four DoMAP levels.

Low intake emerged as the most prominent of the core mechanisms contributing to malnutrition in our hospitalized cohort, appearing in 89% of malnourished inpatients compared with 20% showing increased requirements and 12% with reduced bioavailability. Although 49% of well-nourished patients also reported decreased intake, the prevalence was markedly lower than in the malnourished group. In the regression analysis, low intake was associated with a seven-fold greater likelihood of malnutrition, underscoring its pivotal role among the central drivers. This inadequate consumption often arises from age-related changes such as diminished appetite as well as disease-related symptoms like nausea, oral pain, and inflammation or infection, all of which were significantly correlated with low intake in our cohort. Because reduced intake is itself a core criterion of the GLIM definition, its strong association with malnutrition is partly inherent to the diagnostic framework, while also underscores the clinical importance of early efforts to support adequate energy and intake in acutely ill older adults. Maintaining sufficient nutritional intake is essential not only for immediate recovery but also for long-term functional independence and quality of life in this vulnerable population [2].

In addition to low intake, we observed that malnourished patients had lost nearly 8 kg of body weight prior to their hospital stay, indicating that substantial weight loss had already occurred before admission to the geriatric ward and before the first diagnosis of malnutrition. However, this weight loss occurred predominantly within the last 3 months, demonstrating the contribution of acute disease in this cohort. In addition, early weight loss during hospitalization may further aggravate pre-existing nutritional deficits and has important clinical implications, underscoring the need for close monitoring of body weight and timely nutritional interventions in older hospitalized patients.

Within Level 2 of the DoMAP model, poor appetite was the most prominent determinant, affecting 68% of malnourished patients versus 25% of non-malnourished patients. In the regression analysis, poor appetite was associated with nearly a six-fold increase in the likelihood of malnutrition, underscoring its importance as a key determinant. This observation aligns with prior research. For example, a systematic review of prospective studies in older adults identified poor appetite—alongside hospitalization, eating dependency, and impaired physical function—as a central determinant of malnutrition [8]. Although most studies in this field, including ours, are cross-sectional and therefore do not establish causality, evidence from longitudinal data also supports this association. Specifically, a systematic review reported that among three prospective studies, two longitudinal community-based cohorts found that appetite loss was associated with a 76% increased risk of unintentional weight loss [19] and a 63% increased risk of developing malnutrition [20]. The third cross-sectional study, conducted among 317 orthopedic inpatients, found that those reporting appetite loss had 4.5 times higher odds of being malnourished compared to those without appetite loss (OR = 4.54; 95% CI: 2.31–8.90; *P* < 0.001) [21]. Moreover, both acute and chronic inflammation have been shown to suppress appetite and reduce energy and protein intake [22]. In our cohort, this relationship was evident in the significant correlations between appetite loss and both inflammation and inflammatory disease. Previous research in older hospitalized patients have confirmed that elevated inflammatory markers such as C-reactive protein and pro-inflammatory cytokines are strongly linked to poor appetite and low food intake [23–25]. In addition, inappetence during hospitalization is likely multifactorial, reflecting the combined effects of acute illness, inflammatory responses, medication-related side effects, environmental factors, and temporary fasting for diagnostic or therapeutic procedures.

Analysis of Level 3 determinants identified gastrointestinal disease and oral pain as the most significant determinants associated with malnutrition, while, however, oral pain was only prevalent in 9 patients with malnutrition. Extensive research links deteriorating oral health to poor nutritional status/malnutrition in older adults [26–28], and several systematic reviews have confirmed this relationship [29–31]. Older adults are particularly susceptible to oral health issues including oral pain, periodontal disease, and tooth loss, which compromise chewing efficiency and food selection, leading to poor nutritional status and risk of malnutrition [10]. In a descriptive, cross-sectional study in institutionalized frail elderly, Soini et al. demonstrated that the prevalence of malnutrition rises steadily with the increasing number of oral health problems such as oral pain, chewing difficulties, and xerostomia [32]. Consistent with these findings, we observed a clear association between oral pain, chewing problems and low intake in our hospitalized patients, further supporting the link between oral health and nutritional status.

Notably, dry mouth and sensory impairment were unexpectedly more prevalent among non-malnourished patients, and both were inversely associated with malnutrition in the regression analysis, despite traditionally being viewed as risk factors for low intake. Several factors may explain this paradox. First, dry mouth may be a driver of additional oral intake to ameliorate the sensation of dry mouth, potentially preventing nutritional decline. Second, in the regression model including stronger predictors like gastrointestinal disease, oral pain, or poor appetite, the independent contribution of dry mouth and sensory impairment may be overshadowed or appear reversed. Thus, these inverse relationships likely reflect complex clinical situations and statistical interactions, and should not be interpreted as protective factors. In addition, under-recognition of subjective symptoms such as dry mouth or sensory impairment cannot be excluded, particularly in patients with cognitive impairment or dementia, in whom self-reporting may be limited. However, MoCA scores did not differ significantly between malnourished and non-malnourished patients in our cohort, suggesting that differences in global cognitive status alone are unlikely to fully explain this finding. This may nevertheless have contributed in part to the unexpectedly lower prevalence of these determinants in malnourished patient.

Our study also identified gastrointestinal disease as a significant risk factor for malnutrition in this hospital population, confirming previous evidence and physiological plausibility that gastrointestinal symptoms reduce intake and impaired gastrointestinal function hinders nutrient absorption and compromises overall health [33, 34]. In our cohort, symptoms indicative of gastrointestinal dysfunction such as malabsorption, diarrhea, nausea, and vomiting were markedly more prevalent among malnourished than non-malnourished patients. Although these symptoms did not retain independent significance in the regression model, their high frequency highlights that malnutrition in older hospitalized patients reflects not only the presence of single underlying diseases but also the cumulative burden of multiple clinical manifestations.

Interestingly, certain determinants from Levels 2 and 3 of the DoMAP model, previously shown to be associated with nutritional decline, did not emerge as independent determinants of malnutrition in our analysis. For instance, dysphagia has been identified as a significant contributor to malnutrition in earlier cross-sectional studies [35, 36], yet it was not independently associated with malnutrition in the regression model. This may be partially explained by its low overall prevalence (6%) in our cohort, which limits its statistical impact.

Moreover, cognitive impairment and dementia, although frequently implicated in malnutrition risk, did not differ significantly between malnourished and non-malnourished patients in our study. However, both were strongly correlated with forgetting to eat, suggesting an indirect influence on nutritional status through impaired eating behavior rather than direct physiological mechanisms. These findings highlight the importance of considering intermediate pathways and interactions among determinants, particularly in older individuals where multifactorial dynamics are common.

Previous hospitalization was another significant determinant of malnutrition in our analysis and was more frequent in malnourished than in non-malnourished patients. Prior studies report that 20–50% of older adults are already malnourished at admission [37, 38], nearly half of those hospitalized for more than a week remain malnourished or deteriorate further [39], and about one third of previously well-nourished patients develop malnutrition during their stay [38]. In a retrospective cohort of 470 geriatric inpatients, malnutrition risk was associated with a 54% higher likelihood of prolonged hospitalization (OR 1.54; 95% CI 1.01–2.36) [40]. Several hospital-related factors likely drive this association, including illness-related appetite loss, fasting for diagnostic procedures, medication side effects, gastrointestinal dysfunction, and suboptimal nutritional management. In our cohort, hospitalization was significantly linked to low intake, poor appetite, infection, inflammatory disease, polypharmacy, and multimorbidity, indicating that the hospital environment can intensify multiple interacting risk factors for malnutrition. At the same time, the independent effect of hospitalization itself on nutritional status is difficult to separate from the impact of the acute and chronic diseases that led to admission, as these conditions are often closely interrelated with inflammatory burden, reduced intake, and increased metabolic stress.

From a clinical perspective, our findings emphasize the importance of systematically assessing appetite and actual food intake in older hospitalized patients using standardized screening approaches. Moreover, determinants that showed significant associations with malnutrition in our DoMAP-based analyses may help identify patients at higher risk and guide a focused evaluation for potentially modifiable contributors. Given the strong association between poor appetite and malnutrition, appetite loss should trigger assessment for inflammatory conditions, gastrointestinal disorders, medication-related side effects, oral health problems, and psychosocial factors. Importantly, interventions should not focus solely on nutritional supplementation but also address modifiable contributors to reduced intake.

Interestingly, reduced intake was also observed in a substantial proportion of non-malnourished patients. This finding may reflect early or transient reductions in intake that have not yet resulted in sufficient weight loss or phenotypic criteria to meet the GLIM definition of malnutrition. It therefore suggests that low intake represents an early warning signal rather than a feature exclusive to established malnutrition, highlighting the need for preventive strategies before clinically relevant deterioration occurs.

This study has several limitations. First, its cross-sectional design precludes causal inference between the identified determinants and malnutrition. Second, recruitment was limited to geriatric acute care units in three German hospitals, which may introduce selection bias toward frailer patients and limit generalizability to other healthcare settings. Third, malnutrition was diagnosed with the GLIM framework but phenotypic assessment relied on weight loss and BMI only, excluding muscle mass which could not be assessed reliably, which may have led to a certain under detection of malnutrition according to GLIM. Finally, all determinants were counted equally, without considering their severity or duration. Although the study cohort is of reasonable size, 556 subjects may not be sufficient to detect risk factors with low prevalence such as poverty or poor quality of care, which were reported in only 7 and 11 participants, respectively. Future adaptations of the DoMAP model could therefore benefit from approaches that weight individual determinants according to their relative relevance, rather than treating all factors equally. Such weighting, informed by larger datasets, may enhance the model’s discriminative power and clinical applicability. Future research employing longitudinal designs is warranted to further elucidate the causal mechanisms underlying malnutrition in older hospitalized patients. Additional studies should evaluate whether structured, targeted interventions addressing appetite regulation and symptom control can prevent progression from reduced intake to established malnutrition, and whether weighted determinant models improve risk stratification in clinical practice.

Despite these limitations, this study also has notable strengths. It includes a relatively large sample of more than 500 patients recruited from three different German hospitals, thereby increasing the robustness and representativeness of the findings. Furthermore, it provides a comprehensive view of malnutrition determinants based on the DoMAP framework, which was developed through international expert consensus and thus ensures conceptual rigor. Finally, the use of standardized definitions for determinants supported systematic assessment and comparability across sites, even though some variability between examiners cannot be fully excluded.

## Conclusion

This study underscores the multifactorial nature of malnutrition in older hospitalized patients, showing that both underlying diseases and their accompanying symptoms are critical to its development. Using the DoMAP model, we identified low intake as the dominant core mechanism, with poor appetite, gastrointestinal disease, inflammatory disease, and previous hospitalization among the most important associated determinants in this cohort.

## Supplementary Information

Supplementary Material 1

Supplementary Material 2

Supplementary Material 3

Supplementary Material 4

## Declarations

### Data availability

The datasets analyzed during the current study are available from the corresponding author on reasonable request.
