# Supplementary material for: Determinants of malnutrition in older hospitalized patients: a prospective multicenter study with the DoMAP model
Source: BMC Geriatr. 2026 May 7;26:650. doi: 10.1186/s12877-026-07612-6 (PMC13154458; doi:10.1186/s12877-026-07612-6)
Supplement: Supplementary file 1 — Supplementary Material 1 [file 12877_2026_7612_MOESM1_ESM.docx]

**Supplementary Table 1.** Level 1 of determinants of malnutrition

| Level 1 | Total population  (n=556) | *Non-malnourished  (n=319) | Malnourished  (n=237) | **P value |
| --- | --- | --- | --- | --- |
| Reduced nutrient bioavailability (n, %) |  |  |  |  |
| No | 515 (93) | 306 (96) | 209 (88) | <0.001 |
| Yes | 41 (7) | 13 (4) | 28 (12) |  |
| Low intake (n, %) |  |  |  |  |
| No | 189 (34) | 162 (51) | 27 (11) | <0.001 |
| Yes | 367 (66) | 157 (49) | 210 (89) |  |
| High requirements (n, %) |  |  |  |  |
| No | 486 (88) | 297 (93) | 189 (80) | <0.001 |
| Yes | 68 (12) | 21 (7) | 47 (20) |  |

*Malnutrition was diagnosed based on the Global Leadership Initiative on Malnutrition (GLIM) criteria; **Difference between malnourished and non-malnourished participants
