# Supplementary material for: Determinants of malnutrition in older hospitalized patients: a prospective multicenter study with the DoMAP model
Source: BMC Geriatr. 2026 May 7;26:650. doi: 10.1186/s12877-026-07612-6 (PMC13154458; doi:10.1186/s12877-026-07612-6)
Supplement: Supplementary file 2 — Supplementary Material 2 [file 12877_2026_7612_MOESM2_ESM.docx]

**Supplementary Table 2.** Level 2 of determinants of malnutrition

| Level 2 | Total population  (n=556) | *Non-malnourished  (n=319) | Malnourished  (n=237) | **P value |
| --- | --- | --- | --- | --- |
| Malabsorption |  |  |  |  |
| No | 519 (94) | 306 (96) | 213 (90) | 0.007 |
| Yes | 35 (6) | 13 (4) | 23 (10) |  |
| Diarrhea |  |  |  |  |
| No | 516 (93) | 303 (95) | 213 (90) | 0.030 |
| Yes | 40 (7) | 16 (5) | 23 (10) |  |
| Nausea |  |  |  |  |
| No | 499 (90) | 302 (95) | 197 (83) | <0.001 |
| Yes | 57 (10) | 17 (5) | 40 (17) |  |
| Vomiting |  |  |  |  |
| No | 532 (96) | 315 (99) | 217 (92) | <0.001 |
| Yes | 23 (4) | 4 (1) | 19 (8) |  |
| Tremor |  |  |  |  |
| No | 529 (95) | 304 (95) | 225 (95) | 0.845 |
| Yes | 27 (5) | 15 (5) | 12 (5) |  |
| Dysphagia |  |  |  |  |
| No | 522 (94) | 302 (95) | 220 (93) | 0.377 |
| Yes | 34 (6) | 17 (5) | 17 (7) |  |
| Chewing problems |  |  |  |  |
| No | 533 (96) | 308 (97) | 225 (95) | 0.392 |
| Yes | 23 (4) | 11 (3) | 12 (5) |  |
| Lack of food |  |  |  |  |
| No | 547 (98) | 318 (100) | 229 (97) | 0.006 |
| Yes | 9 (2) | 1 (0) | 8 (3) |  |
| Difficulties with shopping |  |  |  |  |
| No | 426 (77) | 251 (79) | 175 (74) | 0.189 |
| Yes | 130 (23) | 68 (21) | 62 (26) |  |
| Poor appetite |  |  |  |  |
| No | 315 (57) | 238 (75) | 77 (32) | <0.001 |
| Yes | 241 (43) | 81 (25) | 160 (68) |  |
| Restrictive diet |  |  |  |  |
| No | 522 (94) | 302 (95) | 220 (93) | 0.365 |
| Yes | 33 (6) | 16 (5) | 17 (7) |  |
| Unwilling to eat |  |  |  |  |
| No | 466 (84) | 279 (88) | 187 (79) | 0.007 |
| Yes | 90 (16) | 40 (12) | 50 (21) |  |
| Forgetting to eat |  |  |  |  |
| No | 459 (83) | 258 (81) | 201 (85) | 0.259 |
| Yes | 97 (17) | 61 (19) | 36 (15) |  |
| Hyperactivity |  |  |  |  |
| No | 552 (99) | 318 (100) | 234 (99) | 0.317 |
| Yes | 4 (1) | 1 (0) | 3 (1) |  |
| Inflammation |  |  |  |  |
| No | 422 (76) | 259 (81) | 163 (69) | <0.001 |
| Yes | 134 (24) | 60 (19) | 74 (31) |  |
| Increased metabolic rate |  |  |  |  |
| No | 414 (93) | 306 (96) | 208 (88) | <0.001 |
| Yes | 41 (7) | 13 (4) | 28 (12) |  |

*Malnutrition was diagnosed based on the Global Leadership Initiative on Malnutrition (GLIM) criteria; **Difference between malnourished and non-malnourished participants
