# Supplementary material for: Determinants of malnutrition in older hospitalized patients: a prospective multicenter study with the DoMAP model
Source: BMC Geriatr. 2026 May 7;26:650. doi: 10.1186/s12877-026-07612-6 (PMC13154458; doi:10.1186/s12877-026-07612-6)
Supplement: Supplementary file 3 — Supplementary Material 3 [file 12877_2026_7612_MOESM3_ESM.docx]

**Supplementary Table 3.** Level 3 of determinants of malnutrition

| Level 3 | Total population  (n=556) | *Non-malnourished  (n=319) | Malnourished  (n=217) | **P value |
| --- | --- | --- | --- | --- |
| Gastrointestinal disease |  |  |  |  |
| No | 452 (81) | 283 (89) | 169 (71) | <0.001 |
| Yes | 104 (19) | 36 (11) | 68 (29) |  |
| Medication |  |  |  |  |
| No | 287 (52) | 161 (51) | 126 (53) | 0.549 |
| Yes | 269 (48) | 158 (49) | 111 (47) |  |
| Cancer |  |  |  |  |
| No | 497 (89) | 291 (91) | 206 (87) | 0.125 |
| Yes | 59 (11) | 28 (8) | 31 (13) |  |
| Parkinson disease |  |  |  |  |
| No | 521 (94) | 301 (94) | 220 (93) | 0.484 |
| Yes | 35 (6) | 18 (6) | 17 (7) |  |
| Stroke |  |  |  |  |
| No | 489 (88) | 276 (87) | 213 (90) | 0.291 |
| Yes | 66 (12) | 42 (13) | 24 (10) |  |
| Dry mouth |  |  |  |  |
| No | 460 (83) | 249 (78) | 211 (89) | 0.001 |
| Yes | 94 (17) | 68 (22) | 26 (11) |  |
| Poor dental state |  |  |  |  |
| No | 490 (88) | 276 (86) | 214 (90) | 0.187 |
| Yes | 66 (12) | 43 (14) | 23 (10) |  |
| Oral pain |  |  |  |  |
| No | 540 (97) | 312 (98) | 228 (96) | 0.193 |
| Yes | 15 (3) | 6 (2) | 9 (4) |  |
| Pain |  |  |  |  |
| No | 286 (51) | 154 (48) | 132 (56) | 0.087 |
| Yes | 270 (49) | 165 (52) | 105 (44) |  |
| Physical inactivity |  |  |  |  |
| No | 256 (46) | 152 (48) | 104 (44) | 0.438 |
| Yes | 299 (54) | 167 (52) | 132 (56) |  |
| Loneliness |  |  |  |  |
| No | 327 (59) | 192 (60) | 135 (57) | 0.486 |
| Yes | 229 (41) | 127 (40) | 102 (43) |  |
| Poverty |  |  |  |  |
| No | 548 (99) | 318 (100) | 230 (97) | 0.046 |
| Yes | 7 (1) | 1 (0) | 6 (3) |  |
| Poor quality of care |  |  |  |  |
| No | 545 (98) | 316 (99) | 229 (97) | 0.062 |
| Yes | 11 (2) | 3 (1) | 8 (3) |  |
| Poor quality of meals |  |  |  |  |
| No | 530 (95) | 309 (97) | 221 (93) | 0.066 |
| Yes | 26 (5) | 10 (3) | 16 (7) |  |
| Sensory impairment |  |  |  |  |
| No | 492 (88) | 272 (85) | 220 (93) | 0.007 |
| Yes | 64 (12) | 47 (15) | 17 (7) |  |
| Mobility limitations |  |  |  |  |
| No | 181 (33) | 102 (32) | 79 (33) | 0.784 |
| Yes | 375 (67) | 217 (68) | 158 (67) |  |
| Loss of interest in life |  |  |  |  |
| No | 531 (96) | 308 (97) | 223 (94) | 0.140 |
| Yes | 24 (3) | 10 (3) | 14 (6) |  |
| Depression |  |  |  |  |
| No | 455 (82) | 265 (83) | 190 (80) | 0.436 |
| Yes | 101 (18) | 54 (17) | 47 (20) |  |
| Cognitive impairment |  |  |  |  |
| No | 244 (44) | 139 (44) | 105 (44) | 0.863 |
| Yes | 312 (56) | 180 (56) | 132 (56) |  |
| Dementia |  |  |  |  |
| No | 390 (70) | 222 (70) | 168 (71) | 0.779 |
| Yes | 166 (30) | 97 (30) | 69 (29) |  |
| Surgery |  |  |  |  |
| No | 416 (75) | 241 (75) | 175 (74) | 0.693 |
| Yes | 140 (25) | 78 (25) | 62 (26) |  |
| COPD |  |  |  |  |
| No | 490 (88) | 291 (91) | 199 (84) | 0.011 |
| Yes | 66 (12) | 28 (9) | 38 (16) |  |
| Infection |  |  |  |  |
| No | 489 (88) | 287 (90) | 202 (85) | 0.113 |
| Yes | 67 (12) | 32 (10) | 35 (15) |  |
| Inflammatory disease |  |  |  |  |
| No | 419 (76) | 258 (81) | 161 (68) | <0.001 |
| Yes | 134 (24) | 59 (19) | 75 (32) |  |
| Psychological stress |  |  |  |  |
| No | 476 (86) | 273 (86) | 203 (86) | 1.000 |
| Yes | 79 (14) | 45 (14) | 34 (14) |  |

*Malnutrition was diagnosed based on the Global Leadership Initiative on Malnutrition (GLIM) criteria; **Difference between malnourished and non-malnourished participants
