# Supplementary material for: Determinants of malnutrition in older hospitalized patients: a prospective multicenter study with the DoMAP model
Source: BMC Geriatr. 2026 May 7;26:650. doi: 10.1186/s12877-026-07612-6 (PMC13154458; doi:10.1186/s12877-026-07612-6)
Supplement: Supplementary file 4 — Supplementary Material 4 [file 12877_2026_7612_MOESM4_ESM.docx]

**Supplementary Table 4.** Level 4 of determinants of malnutrition

| Level 4 | Total population  (n=556) | *Non-malnourished  (n=319) | Malnourished  (n=237) | **P value |
| --- | --- | --- | --- | --- |
| Polypharmacy |  |  |  |  |
| No | 113 (20) | 66 (21) | 47 (20) | 0.832 |
| Yes | 443 (80) | 253 (79) | 190 (80) |  |
| Multimorbidity |  |  |  |  |
| No | 115 (20) | 65 (20) | 50 (21) | 0.833 |
| Yes | 439 (80) | 253 (80) | 186 (79) |  |
| Low education |  |  |  |  |
| No | 549 (99) | 318 (100) | 231 (97) | 0.046 |
| Yes | 7 (1) | 1 (0) | 6 (3) |  |
| Anorexia of aging |  |  |  |  |
| No | 535 (96) | 318 (100) | 217 (92) | <0.001 |
| Yes | 21 (4) | 1 (0) | 20 (8) |  |
| Age-related functional decline |  |  |  |  |
| No | 437 (79) | 256 (80) | 181 (76) | 0.296 |
| Yes | 119 (21) | 63 (20) | 56 (24) |  |
| Frailty |  |  |  |  |
| No | 278 (50) | 170 (53) | 109 (46) | 0.103 |
| Yes | 277 (50) | 149 (47) | 128 (54) |  |
| Hospitalization |  |  |  |  |
| No | 259 (47) | 168 (53) | 91 (38) | <0.001 |
| Yes | 296 (53) | 150 (47) | 146 (62) |  |

*Malnutrition was diagnosed based on the Global Leadership Initiative on Malnutrition (GLIM) criteria; **Difference between malnourished and non-malnourished participants
